# Association of LEPR Gene Polymorphisms With Youth-Onset Diabetes in Bangladesh

**DOI:** 10.7759/cureus.84696

**Published:** 2025-05-23

**Authors:** Md. Shayedat-Ullah, Nusrat Sultana, Mashfiqul Hasan, U.S. Mahzabin Amin, Tahaia Anan Rahman, Indira Roy, Mukul Rayhan, Palash Chandra Sutradhar, Md. Salimullah, Muhammad Abul Hasanat

**Affiliations:** 1 Endocrinology, Bangladesh Medical University, Dhaka, BGD; 2 Molecular Biotechnology Division, National Institute of Biotechnology, Dhaka, BGD

**Keywords:** bangladesh, leptin receptor gene, single-nucleotide polymorphism, type-2 diabetes mellitus, youth-onset type 2 diabetes

## Abstract

Introduction

Polymorphisms of the leptin receptor (LEPR) gene are associated with type 2 diabetes mellitus (T2DM), but the association varies among different geographic populations. The present study aims to observe the association of single-nucleotide polymorphisms (SNPs) of the LEPR gene (rs1137100 and rs1137101) with youth-onset T2DM in Bangladesh.

Methods

This case-control study encompassed 62 individuals with youth-onset T2DM (age range 18-29 years) and an equal number of age-matched controls with normal glucose tolerance (NGT). Genotyping was done by polymerase chain reaction-restriction fragment length polymorphism (PCR-RFLP). Genotypes of both LEPR SNPs were expressed as AA, AG, and GG, where G is considered a risk allele.

Results

The frequency of G-allele was higher in DM than in NGT for both rs1137100 (55.6% (69/124) vs. 42.7% (53/124); OR 1.7, 95% CI 1.02-2.78; p=0.042) and rs1137101 (59.7% (74/124) vs. 41.9% (52/124); OR 95% CI 1.24-3.40, p=0.005). In the codominant model, the GG genotype was associated with DM (GG vs. AA: rs1137100: OR 3.37; CI 1.11-10.19; p=0.032; rs1137101: OR 4.93; CI 1.62-14.99; p=0.005) but not the AG genotype (AG vs. AA). In the dominant model, the risk variants AG+GG (vs. AA) of rs1137100 did not have an association (p=0.289), but rs1137101 had (OR 2.60; CI 1.07-6.33; p=0.035). In the recessive model, risk variant GG (vs. AG+AA) of both SNPs had an association with DM (rs1137100: OR 2.98; CI 1.19-7.47; p=0.017; rs1137101: OR 3.02; CI 1.25-7.27; p=0.014). No association was significant in any models when adjusted for body mass index (BMI).

Conclusion

Although the LEPR gene SNPs rs1137100 and rs1137101 show a potential association with an increased risk of youth-onset T2DM in the Bangladeshi population, this association appears to be BMI-dependent.

## Introduction

The rising prevalence of type 2 diabetes mellitus (T2DM) is a global public health concern not only for the middle-aged and elderly but also for young adults. There is strong evidence that the condition is increasing among young adults, along with the rising trend of obesity observed in children and adolescents [1]. In a registry of individuals with youth-onset diabetes in India, T2DM has been reported in nearly one-quarter of young people (age <25 years) [2]. Diabetes and impaired fasting glucose (IFG) were found to be prevalent in 1.8% and 3.4% of Bangladeshi children, respectively, with a higher prevalence in urban children with high family incomes [3].

'Youth-onset diabetes' is a term introduced to describe a distinct type of diabetes characterized by the onset of diabetes at a younger age (below 30 years) with a low body mass index (BMI), requiring a high dose of insulin for metabolic control and is usually ketosis-resistant even when insulin is withdrawn [4]. However, in certain ethnicities, BMI is not a good marker of obesity, and insulin resistance prevails in people with relatively low BMI, making them susceptible to T2DM. It has been postulated that T2DM in young people results from complex interactions between genetic, epigenetic, and environmental factors [5,6]. Amongst all the factors, obesity is most closely linked with the rising trend of early-onset T2DM in genetically susceptible younger populations of the world [7].

It is now known that adipose tissue is an active endocrine organ that secretes several adipokines (such as leptin, adiponectin, resistin, etc.) [8]. Obesity-related adipokine dysregulation contributes to insulin resistance, T2DM, and metabolic syndrome [9,10]. Among the adipokines, leptin, a peptide of 167 amino acids found in white adipose tissue, plays a vital role in fatty acid and glucose metabolism. Leptin may have anti-diabetic effects independent of regulating body weight and energy intake by influencing peripheral insulin sensitivity through central nervous system (CNS) mechanisms [11]. It increases AMP-activated protein kinase (AMPK) phosphorylation and improves insulin sensitivity in skeletal muscle by activating PI3K in the hypothalamus [12].

Leptin exerts its effects by binding to specific leptin receptors (LEPRs) in the CNS. The LEPR gene is located on chromosome 1p31, has 20 exons, and spans about 100 kb [13]. Genetic variation at the LEPR locus has been suggested to play a significant role in T2DM and obesity [14,15]. Common single-nucleotide polymorphisms (SNPs) in LEPR, including Lys109Arg (rs1137100), Gln223Arg (rs1137101), and Lys656Asn (rs8179183), have been associated with adiposity, an increase in BMI, weight gain, hyperleptinemia, or susceptibility to leptin resistance in various populations [16]. Of those, rs1137101 (Gln223Arg) and rs1137100 (Lys109Arg) have been studied the most [17]. However, association analyses of these two SNPs, independently or as a haplotype, have yielded contradictory results in various populations and sample types [14,17-20].

In a previous work of our group, a higher level of leptin was observed in individuals with youth-onset diabetes in Bangladesh, reflecting a leptin resistance that may be related to LEPR polymorphism [21]. The association of LEPR polymorphisms with youth-onset T2DM has not been well evaluated in the Bangladeshi population. Considering this, the present investigation aimed to examine any association between SNPs of the LEPR gene (rs1137100 and rs1137101) and youth-onset T2DM in the Bangladeshi population.

## Materials and methods

Study design and participants

A case-control study was conducted encompassing 62 participants with youth-onset T2DM and an equal number of healthy controls with normal glucose tolerance (NGT), aged 18-29 years of both sexes, diagnosed based on American Diabetes Association (ADA) criteria for diabetes mellitus (DM) from March 2019 to November 2020 in the Department of Endocrinology, Bangladesh Medical University (BMU), Dhaka [22]. In the DM arm, patients with type 1 diabetes, gestational diabetes, secondary diabetes, acute critical illness, chronic medical conditions like cardiac, hepatic, renal, or thyroid disorders, chronic infections, or malignancy were excluded. Only healthy, age-matched participants with NGT were enrolled in the control arm.

The sample size was calculated using the following formula:

$$n = \{u\sqrt{[(r_1(1-r_1)+r_0(1-r_0))]}+v\sqrt{[2\bar{r}(1-\bar{r})]}\}^2/(r_0-r_1)^2$$

Here, v = z-value of standard normal distribution at 5% level of significance = 1.96; u = z-value of standard normal distribution at 80% statistical power = 0.84; r_0_ = anticipated probability of exposure among cases for rs1137100 =0.86; r_1_ = anticipated probability of exposure among cases for rs1137101 = 0.94; r ® = (r_0_+ r_1_)/2 = 0.9

According to the above formula, the sample size for each group is 220. However, due to resource limitations, the study could encompass 124 participants, including DM and NGT.

Study procedure

Study participants were screened for diabetes by oral glucose tolerance test (OGTT) and assigned to the T2DM or NGT arm. When one arm was saturated with the desired number of participants, screening OGTT was continued to assign participants to the other arm. Four ml of a whole blood sample for genotyping was collected in EDTA-flushed vacutainer tubes and kept at room temperature in a vertical position for 15-20 minutes, followed by preservation in a freezer at -70 °C until genotyping, which was performed later. The laboratory was blinded to case/control status.

Analytical method

The study used a commercial DNA extraction kit (PureLink® Genomic DNA Mini Kit, Invitrogen by Thermo Fisher Scientific, Waltham, MA) for DNA extraction from peripheral blood leukocytes. The step of polymerase chain reaction-restriction fragment length polymorphism (PCR-RFLP) includes DNA isolation from peripheral blood leucocytes, PCR amplification of desired fragments, restriction digestion of PCR products, agarose gel electrophoresis, and estimation of fragment length. Genotypes of LEPR variants (rs1137100 and rs1137101) were expressed as AA, AG, and GG, where G is considered a risk allele. PCR amplification of DNA fragments containing the SNPs of interest was performed using specific primers with the ProFlex PCR System (Applied Biosystems, Life Technologies, Thermofisher Scientific). Restriction digestion of PCR products was done using specific enzymes (HaeIII; MspI; New England Biolabs, Ipswich, MA), followed by agarose gel electrophoresis (1-4%) to determine restriction fragment length. Fragments were then separated and visualized by ethidium bromide staining to determine genotype (Appendix). Six randomly selected samples were re-genotyped using Sanger sequencing to confirm the results. The analysis, conducted with SeqScape software (Applied Biosystems), showed complete concordance between the original and re-genotyped samples.

Ethical aspects

The study was conducted in accordance with the Declaration of Helsinki. Before the commencement of this study, the research protocol was approved by the Institutional Review Board (IRB) of BMU (No. BSMMU/2019-8388). The participants gave informed written consent.

Statistical analysis

Data from the study were analyzed using IBM SPSS Statistics for Windows version 25.0 (IBM Corp, Armonk, NY). Data were expressed in frequencies and percentages for qualitative values and mean (±SD) for quantitative values with normal distribution. Hardy-Weinberg equilibrium analyses were conducted using the chi-squared (χ²) goodness-of-fit test with one degree of freedom, separately for the DM and control groups. The frequency of SNPs between the DM and control groups was compared using the χ2 test within different genetic models (dominant, recessive, co-dominant, and allelic) [23]. Risk estimation of SNPs was expressed through crude odds ratio (OR) with a 95% confidence interval (CI) and adjusted OR (after adjusting for BMI). Clinical variables were compared between DM and NGT by Student’s independent t-test or χ2 test. P-values ≤0.05 were considered statistically significant.

## Results

Participants with DM showed significantly higher BMI, waist circumference (WC), hip circumference (HC), waist-hip ratio (WHtR), systolic blood pressure (SBP), diastolic blood pressure (DBP) (p<0.001 for all), as well as a higher prevalence of acanthosis nigricans (p=0.016), compared to those in the NGT group. At the same time, no significant difference was observed between the two groups for age (p=0.115), gender (p=0.279), positive family history of DM in first-degree relatives (p=0.150), and smoking history (p=0.284) (Table 1).

**Table 1:** Characteristics of the study participants (N=124) Within parentheses are percentages over the column total. Significance values represent comparisons between DM and NGT groups by independent sample t-test, chi-squared, and Fisher’s exact test as applicable. * Family history of DM in first-degree relatives DM: diabetes mellitus, NGT: normal glucose tolerance, HC: hip circumference, BMI: body mass index, WC: waist circumference, WHR: waist-hip ratio, WHtR: waist-height raio, SBP: systolic blood pressure, DBP: diastolic blood pressure

Variables	DM (n=62)	NGT (n=62)	P-value
Age (years±SD)	26.2±3.1	25.2±3.3	0.115
Gender			
Male	31 (50.0)	37 (59.7)	0.279
Female	31 (50.0)	25 (40.3)	
Family history of DM^+^	33 (53.2)	25 (40.3)	0.150
Smoker	10 (16.1)	6 (9.7)	0.284
Acanthosis nigricans	23 (37.1)	11 (17.7)	0.016
WC (cm)			
Male	92.3±6.9	75.2±6.7	<0.001
Female	91.9±7.9	74.8±7.3	<0.001
WHR			
Male	0.94±0.04	0.82±0.08	<0.001
Female	0.92±0.06	0.83±0.04	<0.001
WHtR			
<0.5 (Low risk)	2 (3.2)	46 (74.2)	<0.001
≥0.5 (High risk)	60 (96.8)	16 (25.8)	
BMI (Kg/m^2^)	26.2±3.3	20.5±6.9	<0.001
SBP (mmHg)	120.4±14.1	108.9±12.6	<0.001
DBP (mmHg)	78.1±8.8	71.1±7.6	<0.001

The observed genotype frequencies of both SNPs did not show significant deviation from the Hardy-Weinberg equilibrium (p = NS for both SNPs).

Allele frequency of LEPR rs1137100 (+326A>G) and rs1137101 (+668A>G)

**Figure 1:**
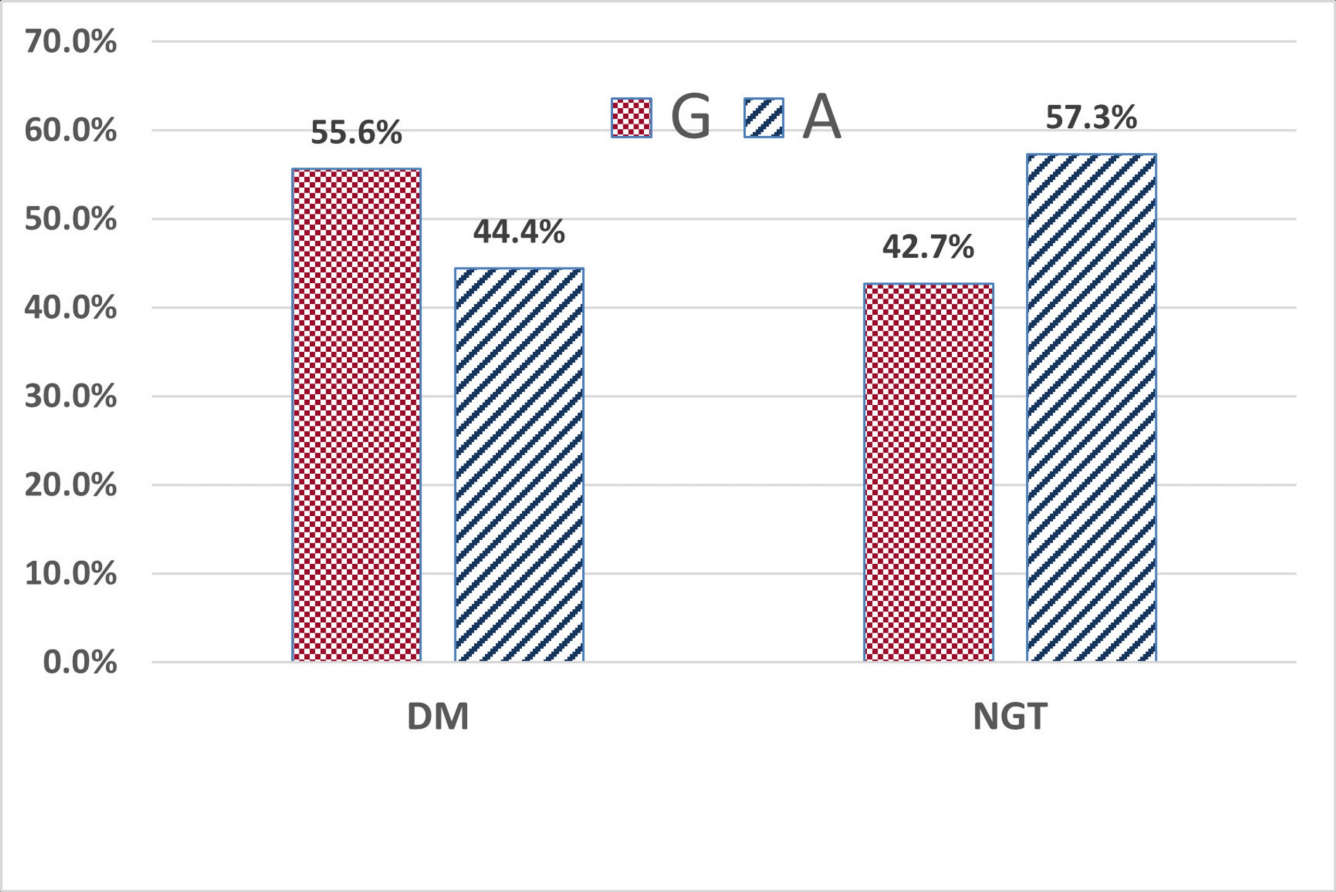
Allele frequency of rs1137100 (+326A>G) in the study participants (N=124) Percentages are over each x-axis category total. Comparison of allele frequency between the DM and NGT groups was performed using the chi-squared test (p = 0.042) T2DM: type 2 diabetes mellitus, NGT: normal glucose tolerance

**Figure 2:**
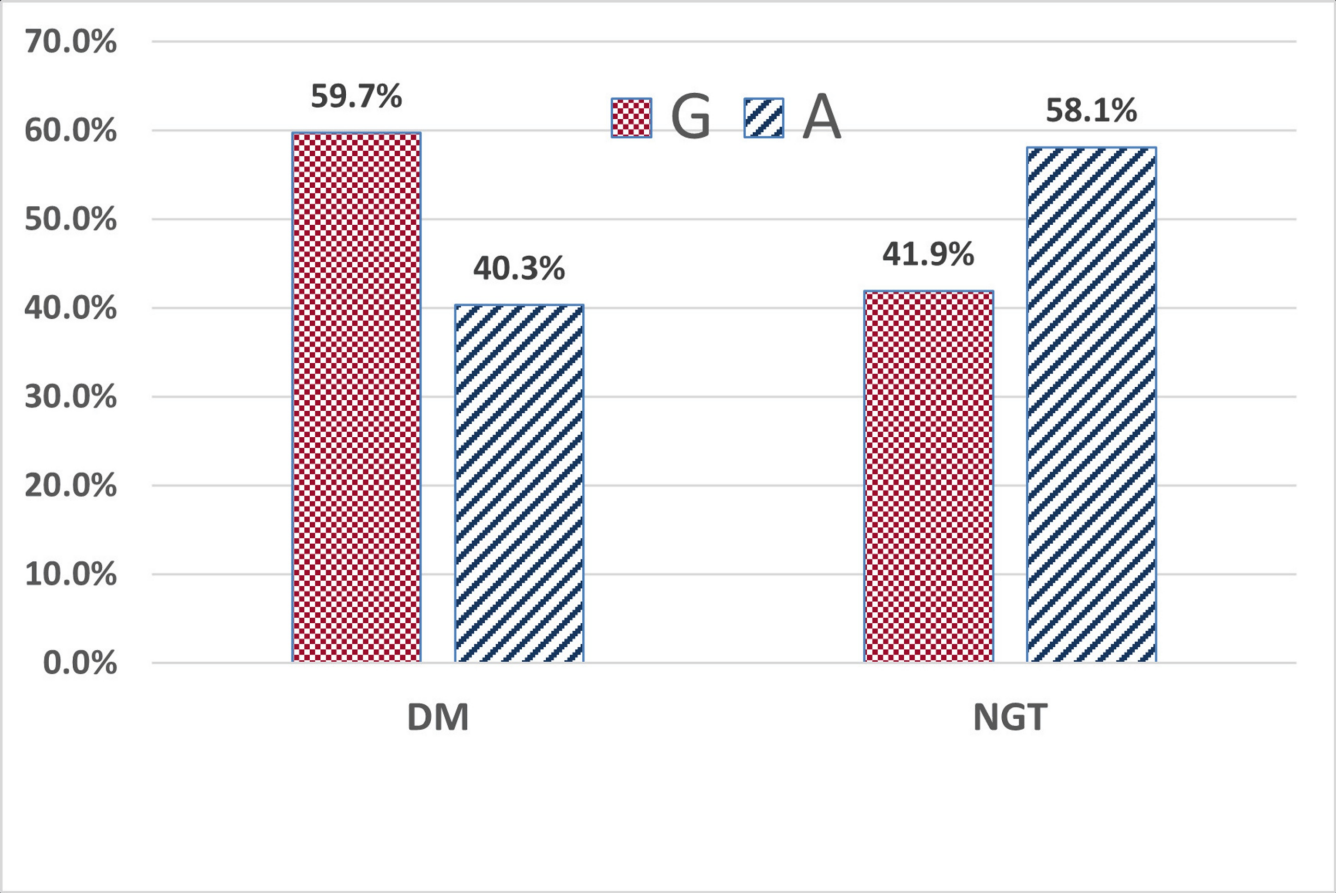
Allele frequency of rs1137101 (+668A>G) in the study participants (N=124) The percentages are over each x-axis category total. Comparison of allele frequency between the DM and NGT groups was performed using the chi-squared test (p = 0.005). T2DM: type 2 diabetes mellitus, NGT: normal glucose tolerance

The risk allele (G) frequency was significantly higher in individuals with youth-onset T2DM for both LEPR rs1137100 and rs1137101 variants (p=0.042, p=0.005, respectively). The carriers of the G allele had 1.7 times (95% CI 1.02-2.78) and 2 times (95% CI 1.24-3.40) higher degree of association with T2DM for rs1137100 and rs1137101 variants, respectively (Figures 1, 2).

Genotype frequencies of LEPR rs1137100 (+326A>G): T2DM vs. NGT

**Figure 3:**
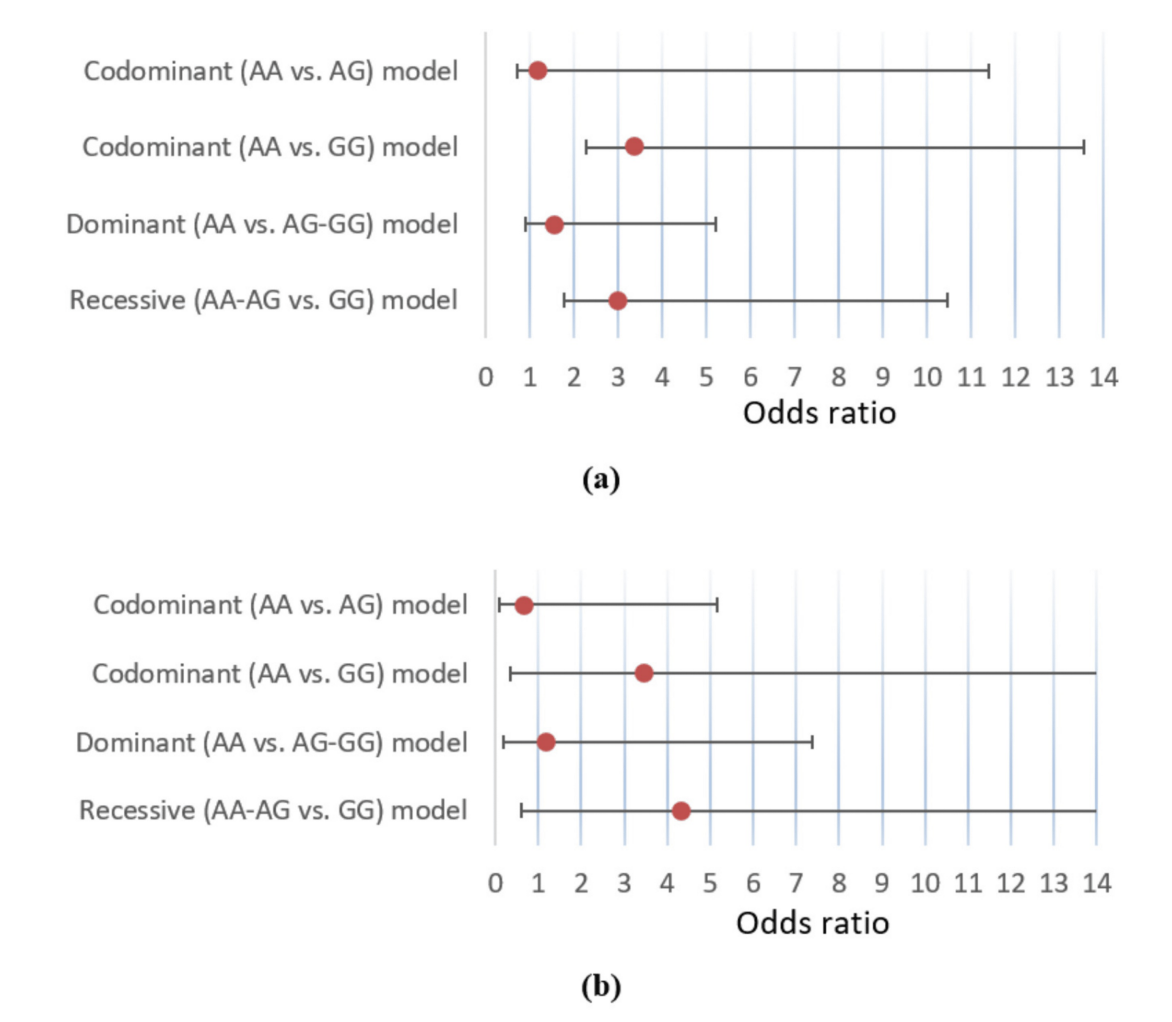
Associations of rs1137100 with youth-onset T2DM under different genetic models (a) unadjusted and (b) adjusted for body mass index, with odds ratios shown by closed circles and whiskers representing the 95% confidence intervals. T2DM: type 2 diabetes mellitus

In the codominant model, the GG genotype was significantly associated with DM (OR 3.37; CI 1.11-10.19; p=0.032) but not AG (p=0.703). In the dominant model, the risk variants (AG+GG) did not have a significant association (p=0.289), whereas, in the recessive model, risk variant GG had a significant association with DM (OR 2.98; CI 1.19-7.47; p=0.017). However, when adjusted for BMI, no association was statistically significant (p=NS for all) (Figure 3).

Genotype frequencies of LEPR rs1137101 (+668A>G): T2DM vs. NGT

**Figure 4:**
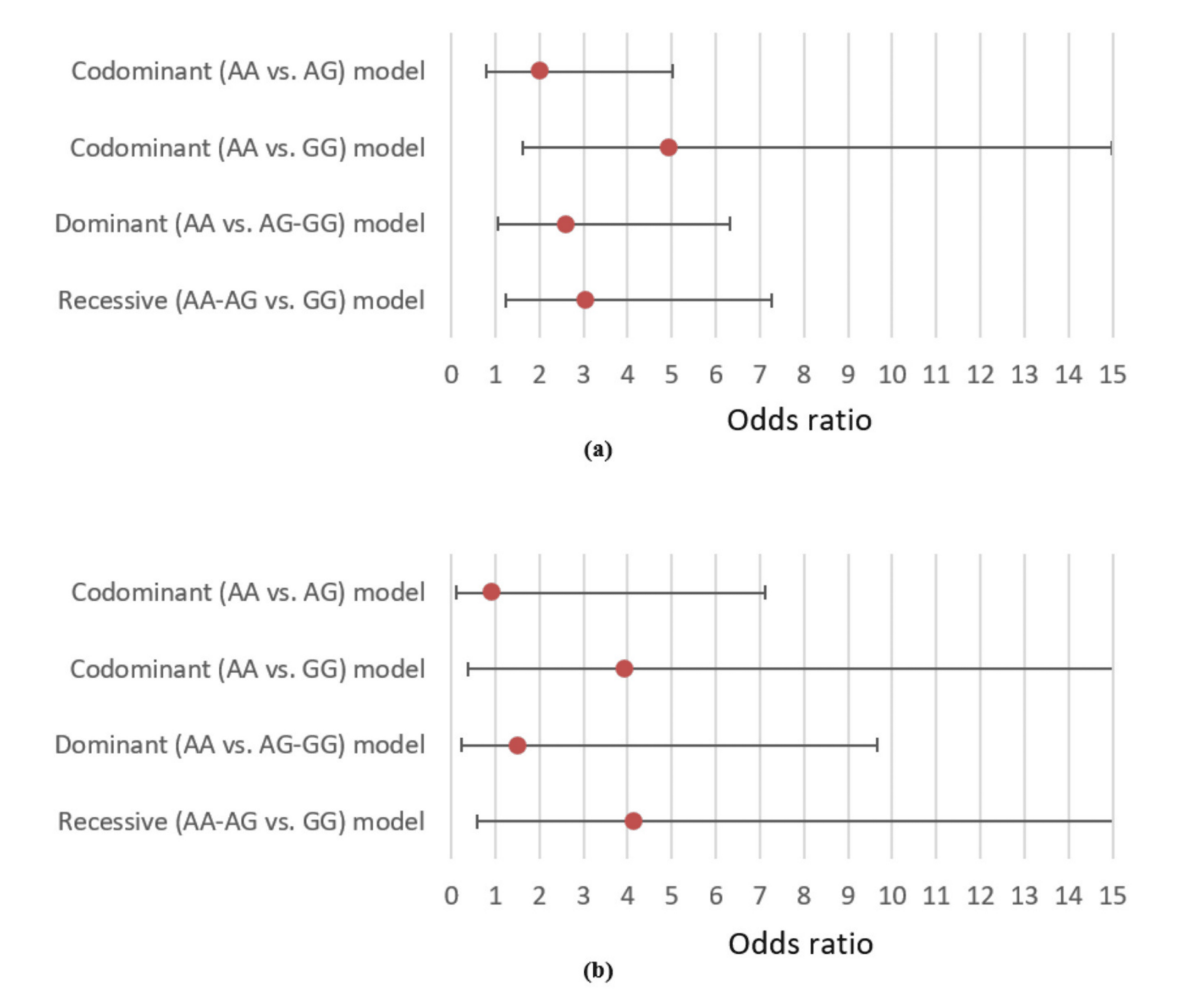
Associations of rs1137101 with youth-onset T2DM under different genetic models (a) unadjusted and (b) adjusted for body mass index, with odds ratios shown by closed circles and whiskers representing the 95% confidence intervals T2DM: type 2 diabetes mellitus

In the codominant model, the GG genotype was significantly associated with DM (OR 4.93; CI 1.62-14.99; p=0.005) but not AG (p=0.147). Both in the dominant and recessive models, the risk variants (GG+AG and GG, respectively) had a significant association with DM (OR 2.60; CI 1.07-6.33; p=0.035 and OR 3.02; CI 1.25-7.27; p=0.014). Again, when adjusted for BMI, no association was statistically significant in any models (p=NS for all) (Figure 4).

## Discussion

The genetic aspects of diabetes in South Asian ethnicity have yet to be explored in the existing literature [24]. A preponderance of the LEPR gene polymorphisms is associated with T2DM, obesity, and metabolic syndrome in different populations [17,21,22,24-26]. The present study observed that two SNPs in LEPR were significantly associated with T2DM in young Bangladeshis. The T2DM group had a significantly higher risk (G) allele frequency than the NGT group for both the LEPR variants. The risk allele imposed 1.6- and 2-times higher degrees of association with T2DM for rs1137100 and rs1137101 variants, respectively. The genotype (GG, AG, and AA) frequencies of rs1137100 varied between T2DM and NGT, where the risk genotype (GG) was higher in the T2DM group in both codominant and recessive models. On the other hand, for the rs1137101 variant, the GG, AG, and AA genotype frequencies varied significantly between T2DM and NGT, with a significant association to the risk genotype (GG) in all (codominant, dominant, and recessive) genetic models. 

These findings suggest that the LEPR gene polymorphisms could affect genetic expression depending on the specific genetic model. The codominant model revealed significant associations with T2DM, indicating that the presence of homozygous or heterozygous genotypes (GG, AG) might contribute differently to the disease’s onset. In contrast, while also showing significant associations, the dominant and recessive models reflect how the genetic risk may vary in individuals depending on whether the risk allele is present in one or both copies of the gene. However, the relationships were not statistically significant when adjusted for BMI.

Previous studies have observed that rs1137100 and rs1137101 SNPs, leading to an A>G nucleotide change, are primarily linked with increased adiposity (BMI and fat mass) [9,20,27]. Additionally, these LEPR polymorphisms have been associated with diabetes, insulin resistance, and metabolic syndrome [17,18,20,21,28]. In the current study, the significant difference in genotype frequencies between young T2DM and control groups highlights the potential role of these SNPs in T2DM development. Polymorphisms in LEPR may lead to altered structure and function of the leptin receptor, impairing the leptin-associated signaling pathway, which, in turn, could inhibit the beneficial effects of leptin and contribute to insulin resistance and T2DM [18]. However, it is important to note that leptin levels were not measured in this study, which may limit the ability to directly correlate these genetic variants with the functional effects of leptin in this population.

The lack of significance when adjusting for BMI in the associations of rs1137100 and rs1137101 may suggest that obesity plays a central role in modulating how these polymorphisms affect the expression of leptin receptors and the subsequent pathophysiological pathways leading to T2DM. Furthermore, environmental factors such as diet, physical activity, and other metabolic factors could influence the expression of these genetic variants, potentially altering their role in the development of T2DM. Future studies incorporating gene-environment interactions will be essential for a more comprehensive understanding of the genetic underpinnings of T2DM in South Asian populations.

This study did not investigate the polymorphism in other loci precipitating T2DM. Thus, it cannot be inferred that findings of glycemic status in the study subjects are solely attributable to the variation of investigated SNPs. The sample size of the current study was also not very large. However, our result reached statistical significance even at this sample size, which may reflect the strong association of LEPR (rs1137100 and rs1137101) gene variants in our study population. Evaluation of a single SNP in a few subjects will unlikely lead to any precise decision. However, this is the first study to evaluate the genetic association of LEPR SNPs and T2DM in the young Bangladeshi population. Given the influence of the variants on youth-onset T2DM, it may be considered an important genome marker for understanding the pathophysiology of T2DM. Answers to these issues lie in future studies.

## Conclusions

In conclusion, our study shows that the LEPR gene rs1137100 and rs1137101 polymorphisms are significantly more frequent in youth-onset T2DM compared to NGT in the Bangladeshi population. However, the associations were not significant when adjusted for BMI, indicating that obesity may influence the genetic risk of T2DM. Leptin levels were not measured in this study, which limits the direct correlation with the genetic variants. Future studies should consider BMI, leptin levels, and multiple polymorphisms of leptin as well as the LEPR gene to better understand their role in T2DM development.

## Figures and Tables

**Figure 5:**
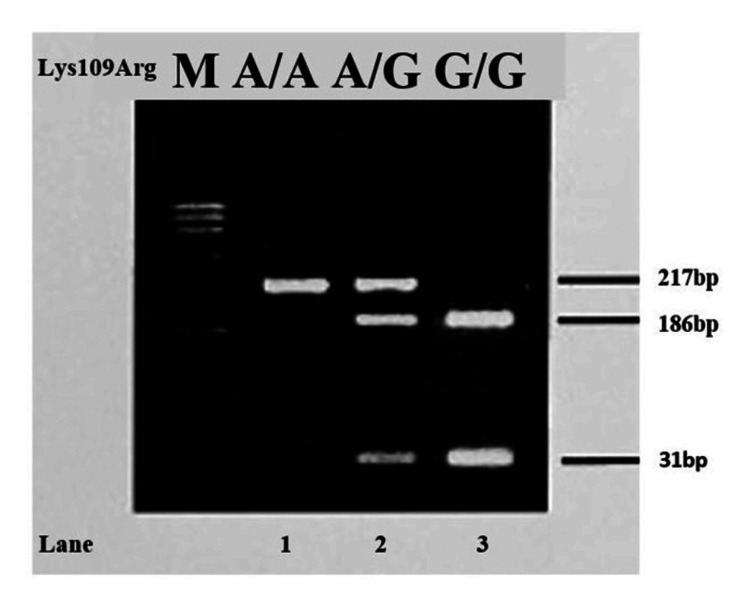
Agarose gel electrophoresis of PCR-RFLP of LPER gene rs1137100 Lane 1 shows homozygous (AA) genotype; Lane 2 shows heterozygous (AG) genotype, and Lane 3 shows homozygous (GG) genotype. PCR-RFLP: polymerase chain reaction-restriction fragment length polymorphism

**Figure 6:**
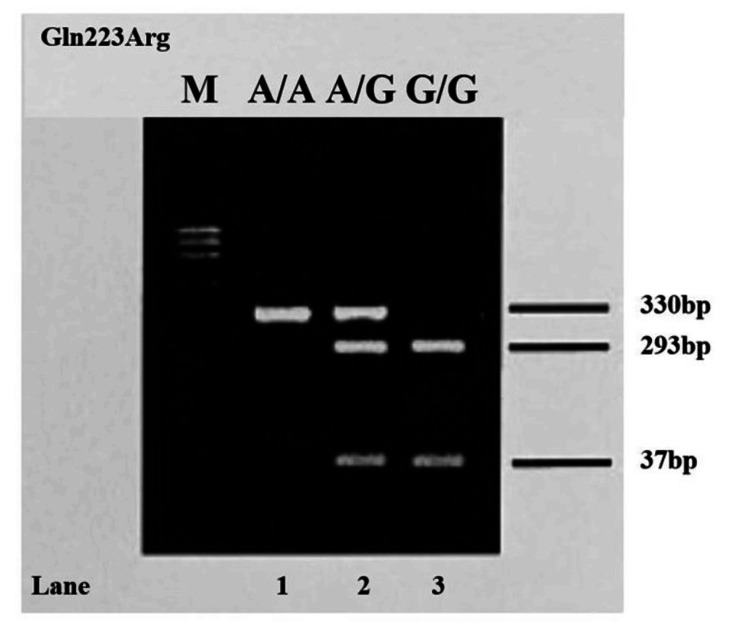
Agarose gel electrophoresis of PCR-RFLP of LPER gene rs1137101 Lane 1 shows homozygous (AA) genotype; Lane 2 shows heterozygous (AG) genotype; Lane 3 shows homozygous (GG) genotype. PCR-RFLP: polymerase chain reaction-restriction fragment length polymorphism

## Appendices

The polymerase chain reaction-restriction fragment length polymorphism (PCR-RFLP) assay method was carried out to determine the distribution of allele and genotype frequencies of the SNP of rs1137100 (+326A>G) and rs1137101 (+668A>G) of LEPR gene using specific primers and restriction enzymes. This was done in Molecular Biotechnology Division of National Institute of Biotechnology (NIB), Savar, Dhaka. The step of PCR-RFLP includes DNA isolation from peripheral blood leucocytes, PCR amplification of desired fragments, restriction digestion of PCR products, agarose gel electrophoresis and estimation of fragment length.

DNA extraction

Commercial DNA extraction kit (PureLink® Genomic DNA Mini Kit, Invitrogen by Thermo Fisher Scientific, USA, Cat No: K1820-01, Lot No: 2164491), which employs silica-membrane-based spin columns for the isolation of genomic DNA from peripheral blood leukocytes, was used in the study. Genomic DNA concentration was determined by spectrophotometry measurement (using NANODROP 2000/2000c, ThermoFisher Scientific). The purity was determined by calculating the ratio of absorbance at 260nm to absorbance at 280 nm (A260/A280). Pure DNA should have an A260/A280 ratio of 1.70-1.90.         

PCR amplification of DNA fragments containing the SNPs of interest

The PCR mix was prepared by including the below-mentioned ingredients, amounting to a total volume of 25.0 ml (Table 2). 

**Table 2:** Ingredients of PCR mix PCR: polymerase chain reaction

Ingredients	Amount
DNA template	1.5 μl
GoTaq® G2 colorless Master Mix (PROMEGA) 10X PCR buffer 50mM MgCl_2_ 1mM dNTPs Taq DNA polymerase	12.5 μl
Forward primer (100ng/ml)	0.5 μl
Reverse primer (100ng/ml)	0.5 μl
Nuclease-free water	10 μl
Total volume	25 μl

Primers used in the PCR are shown in Table 3.

**Table 3:** Primers used for PCR PCR: polymerase chain reaction

SNP	rs1137100 (+326A>G)	rs1137101 (+668A>G)
Forward primer	5 - CTTTTGCCTGCTGGACTCTC-3	5 - ACCCTTTAAGCTGGGTGTCCCAAATAG-3
Reverse primer	5- AAACTAAAGAATTTACTGTTGAAACAAATGTC-3	5 - CAATATTTATGGGCTGAACTGACATT-3

PCR was performed using ProFlex PCR System (applied Biosystems by life technologies) with initial denaturation at 95°C for 5 minutes as a hot start and 95°C for 60 second, 57°C  for 60 second and 72°C for 60 second in 35 cycles of PCR amplification, followed by a final extension step at 72°C for 8 minutes in the presence of  Go Taq G2 Colorless Master Mix (Promega, USA, Cat no: M7832). The resultant PCR products containing SNP rs1137100 (+326A>G) and rs1137101 (+668A>G) were the length of 217 bp and 330 bp, respectively.

Restriction digestion

PCR products (7µl) were then treated with 0.5 µl restriction enzyme (New England Biolabs, USA, HaeIII, Cat no:R0108S; MspI, Cat no: R0106S) and were kept at 37°C overnight (16 hours). Reaction mixture for restriction enzyme analysis of SNP was prepared as follows to a final volume of 10.0 mL (Table 4). 

**Table 4:** Ingredients of reaction mixture for restriction enzyme analysis

Ingredients	Volume
PCR product	7.0 ml
Reaction buffer	1.0 ml
Restriction Enzyme	0.5 ml
PCR H_2_O	1.5 ml
Total Volume	10.0 ml

Agarose gel electrophoresis

Restriction-digested PCR products underwent electrophoresis in a 1-4 % agarose gel with ethidium bromide stain for the determination of restriction fragment length. Fragments were then separated and visualized by ethidium bromide staining, 

Determination of genotype (sequencing)

These were done by analyzing the restriction fragment lengths as shown in Table 5 and Figures 5,6.

**Table 5:** Restriction fragment lengths of different genotypes of targeted SNPs SNP: single-nucleotide polymorphism

rs1137100 (+326A>G)		rs1137101 (+668A>G)	
Genotype	Restriction fragments length	Genotype	Restriction fragments length
AA	217bp	AA	330bp
AG	217bp, 186bp, 31bp	AG	330bp, 293bp, 37bp
GG	186bp, 31bp	GG	293bp, 37bp
